# General versus Neuraxial Anesthesia on Clinical Outcomes in Patients Receiving Hip Fracture Surgery: An Analysis of the ACS NSQIP Database

**DOI:** 10.3390/jcm12113827

**Published:** 2023-06-02

**Authors:** Ming-Tse Wang, Chuen-Chau Chang, Chih-Chung Liu, Yu-Hsuan Fan Chiang, Yu-Ru Vernon Shih, Yuan-Wen Lee

**Affiliations:** 1 Department of Anesthesiology, Taipei Medical University Hospital, Taipei 11031, Taiwan; 2 Department of Anesthesiology, Taitung MacKay Memorial Hospital, Taitung 95054, Taiwan; 3 Department of Anesthesiology, School of Medicine, College of Medicine, Taipei Medical University, Taipei 11031, Taiwan; 4 Department of Orthopaedic Surgery, Duke University, Durham, NC 27708, USA

**Keywords:** hip fracture, anesthesia, postoperative outcomes, propensity score, morbidity, mortality

## Abstract

Whether the use of neuraxial anesthesia or general anesthesia leads to more favorable postoperative outcomes in patients receiving hip fracture surgery remains unclear. We used data from the American College of Surgeons National Surgical Quality Improvement Program (ACS NSQIP) Data Files between 2016 and 2020 to investigate the association of neuraxial anesthesia and general anesthesia with morbidity and mortality after hip fracture surgery. Inverse probability of treatment weighting (IPTW) was used to balance the baseline characteristics, and multivariable Cox regression models were used to estimate the hazard ratio (HR) with a 95% confidence interval (CI) for postoperative morbidity and mortality among the different anesthesia groups. A total of 45,874 patients were included in this study. Postoperative adverse events occurred in 1087 of 9864 patients (11.0%) who received neuraxial anesthesia and in 4635 of 36,010 patients (12.9%) who received general anesthesia. After adjustment for IPTW, the multivariable Cox regressions revealed that general anesthesia was associated with increased risks of postoperative morbidity (adjusted HR, 1.19; 95% CI, 1.14–1.24) and mortality (adjusted HR, 1.09; 95% CI, 1.03–1.16). The results of the present study suggest that, compared with general anesthesia, neuraxial anesthesia is associated with lower risks of postoperative adverse events in patients undergoing hip fracture surgery.

## 1. Introduction

Hip fractures are one of the most common healthcare problems in older adults. The worldwide annual incidence of hip fracture was reported as 1.6 million in 2000, and this incidence is expected to increase to 6.3 million by 2050 [1]. As nonsurgical management is associated with higher mortality and poor functional recovery, surgical repair has become the mainstay treatment for hip fractures [2]. The majority of hip fractures occur in the older adult population; thus, patients with hip fractures are at substantial risk of mortality and cardiovascular, pulmonary, thrombotic, infectious, and bleeding complications, which contribute to tremendous medical costs [3]. The annual cost of treatment for hip fractures was reported to be more than $10 billion in the United States alone [4].

Despite efforts to improve the perioperative care of patients with hip fractures, the postoperative 30-day mortality rate was reported to be 10%, and approximately 20% of patients developed severe postoperative complications [5]. Anesthesia is an essential aspect of multidisciplinary perioperative care, which improves clinical outcomes in patients with hip fractures [6]. The most frequently used anesthesia techniques for hip fracture surgery are general anesthesia and neuraxial anesthesia [7]. Neuraxial anesthesia was reported in a meta-analysis to be associated with a reduced risk of in-hospital mortality, acute respiratory failure, and readmission in older adults undergoing hip fracture surgery [8]. However, the results of a different meta-analysis revealed no significant difference in 30-day mortality and the prevalence of pneumonia, acute myocardial infarction, and renal failure between patients who received neuraxial and those who received general anesthesia during hip fracture surgery [9]. Differences in the definition of outcome, follow-up time, and methodology in previous studies may be reasons for the inconsistent results of these meta-analyses. Therefore, whether the use of neuraxial anesthesia or general anesthesia leads to more favorable postoperative outcomes in patients undergoing surgical repair of hip fractures remains controversial.

The American College of Surgeons National Surgical Quality Improvement Program (ACS NSQIP^®^) is a nationally validated program for measuring and improving the quality of surgical care; the program has compiled high-quality data from more than 600 participating hospitals in the United States and around the world [10]. The ACS NSQIP database contains data based on patients’ medical charts that were collected by trained and certified reviewers, which are more trustworthy than those derived from insurance claims and were used in previous studies. Therefore, this study aimed to use the clinical data from the ACS NSQIP to investigate the associations of neuraxial anesthesia and general anesthesia with postoperative outcomes in patients who received hip fracture surgery and provide optimal anesthesia technique recommendations.

## 2. Materials and Methods

### 2.1. Data Source

The data used in this matched prospective cohort study were collected from the ACS NSQIP database [10]. The ACS NSQIP database contains more than 150 surgical variables for up to 30 days following surgery; the data were collected from patients’ medical charts by trained and certified Surgical Clinical Reviewers. The data from the ACS NSQIP database have been demonstrated to be highly trustworthy, with an inter-reviewer disagreement rate of below 2% [11]. In addition to the essential Participant Use Data File (PUF), Procedure-Targeted PUF datasets, which address specific predictors and outcomes for many types of operations, were also released from the ACS NSQIP database. The ACS NSQIP Hip Fracture Procedure-Targeted PUFs consisting of additional variables specific to hip fracture patients treated with open reduction and internal fixation (Current Procedural Terminology (CPT) codes: 27236, 27244, and 27245) from 2016 to 2020 were also available. Therefore, we used the ACS NSQIP Hip Fracture Procedure-Targeted PUFs to investigate the association between different anesthesia techniques and clinical outcomes in patients receiving hip fracture surgery.

### 2.2. Study Population Selection and Clinical Characteristics

The present study comprised patients in the ACS NSQIP Hip Fracture Procedure-Targeted PUFs between 2016 and 2020; patients who were aged ≥18 years and who received hip fracture surgery with CPT codes 27236, 27244, and 27245 were included. The baseline demographics and comorbidities of the study population were obtained from the essential ACS NSQIP PUFs and Hip Fracture Procedure-Targeted PUF datasets; these included age, sex, race or ethnicity, body mass index, functional health status, smoking, diabetes mellitus, hypertension, congestive heart failure, chronic obstructive pulmonary disease (COPD), dialysis, dementia, cancer, bleeding disorder, type of fracture, American Society of Anesthesiologists (ASA) physical status classification, deep venous thrombosis (DVT) prophylaxis, and type of anesthesia. The patients’ major comorbidities were identified according to the surgeons’ preoperative notes. The definitions of variables in the ACS NSQIP database are available in the NSQIP User Guide [10]. To compare the effects of general anesthesia and neuraxial anesthesia on postoperative clinical outcomes, patients who were administered anesthesia other than general or neuraxial were excluded. Patients who had outcome diagnoses at the time of surgery or who had missing data on baseline characteristics were excluded to prevent confounding factors. In addition, patients with missing data regarding the time of outcome occurrence were excluded.

### 2.3. Study Outcomes

The primary outcome was any postoperative 30-day adverse event, which was a composite outcome including postoperative 30-day morbidity and mortality. The secondary outcomes included postoperative 30-day morbidity and mortality. Postoperative 30-day morbidity consisted of major postoperative adverse events, including myocardial infarction, cardiac arrest, stroke, pneumonia, pulmonary embolism, ventilator support for more than 48 h, acute renal failure or progressive renal insufficiency, surgical site infection, sepsis or septic shock, and DVT [8,9,12]. Detailed definitions of each adverse event can be found in the NSQIP User Guide [10].

### 2.4. Statistical Analysis

The baseline characteristics of the study population were summarized using counts and percentages for both neuraxial and general anesthesia. To balance the baseline characteristics between the different anesthesia groups, inverse probability of treatment weighting (IPTW) based on the propensity score was used [13,14]. The propensity score was defined as the probability that a patient was assigned general anesthesia based on the observed covariates. We estimated the propensity score using a multivariable logistic regression model with all the baseline characteristics listed in Table 1. Using the IPTW approach, each patient was weighted by the inverse of the probability of receiving general anesthesia. This approach created a weighted pseudosample of patients in which the selection of general anesthesia was independent of the baseline characteristics. The standardized mean difference (SMD) was used to compare the baseline characteristics between the neuraxial and general anesthesia groups. An SMD of less than 0.1 was considered a negligible difference between the two groups.

Cox regression models were used to estimate the hazard ratio (HR) with a 95% confidence interval (CI) for clinical outcomes between the different anesthesia groups. Adjusted HRs were calculated after adjustment for age, sex, race or ethnicity, body mass index, functional health status, smoking, diabetes mellitus, hypertension, congestive heart failure, COPD, dialysis, dementia, cancer, bleeding disorder, type of fracture, ASA physical status classification, and DVT prophylaxis.

To evaluate the robustness of our findings, we conducted propensity score matching as a sensitivity analysis. Patients who received general anesthesia and those who received neuraxial anesthesia were matched 1:1 using greedy matching with a caliper width of 0.2 times the standard deviation of the logits of the propensity score [14].

All analyses were performed using the SAS System for Windows 9.4 (SAS Institute, Cary, NC, USA). Statistical significance was determined as a *p* value less than 0.05.

## 3. Results

### 3.1. Study Sample Selection

**Figure 1:**
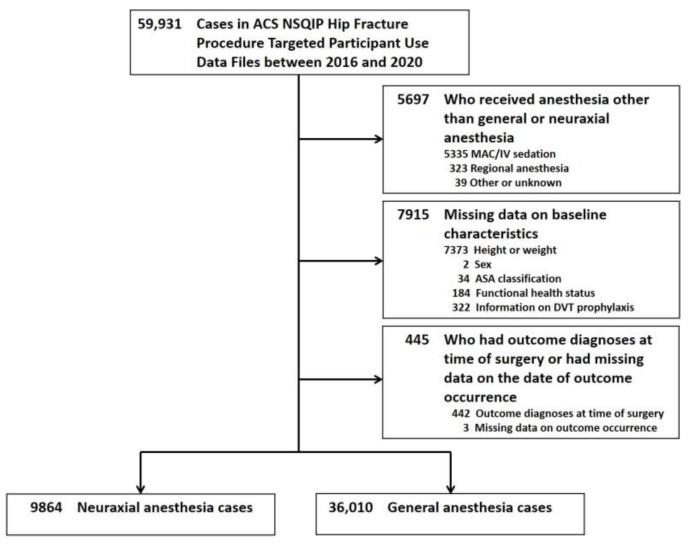
Flowchart of Study Sample Selection. Abbreviations: MAC, monitored anesthesia care; IV, intravenous; ACS NSQIP, American College of Surgeons National Surgical Quality Improvement Program; ASA, American Society of Anesthesiologists; DVT, deep venous thrombosis.

A total of 59,931 cases were reported in the ACS NSQIP Hip Fracture Procedure-Targeted PUFs between 2016 and 2020. After excluding 5697 patients who received anesthesia other than general or neuraxial anesthesia, 7915 patients with missing data on baseline characteristics, and 445 patients who had outcome diagnoses at the time of surgery or had missing data on the date of outcome occurrence, we included a total of 45,874 patients (Figure 1).

### 3.2. Baseline Characteristics

Among the 45,874 patients who received hip fracture surgery, 9864 and 36,010 received neuraxial and general anesthesia, respectively. The baseline characteristics before and after adjustment for IPTW are listed in Table 1. Before adjustment for IPTW, patients who received general anesthesia were generally younger, more likely to be white and obese, and more likely to have bleeding disorders. After adjustment for IPTW, all baseline characteristics of the two groups were well-balanced.

### 3.3. Associations between General Anesthesia and Postoperative Adverse Events

#### 3.3.1. Unweighted Multivariable Analysis

Postoperative adverse events occurred in 1087 of 9864 patients (11.0%) who received neuraxial anesthesia and in 4635 of 36,010 patients (12.9%) who received general anesthesia (Table 2). In the unweighted multivariable regressions, general anesthesia was associated with a 15% increased risk of postoperative adverse events (adjusted HR, 1.15; 95% CI 1.07–1.23) in patients receiving hip fracture surgery (Table 2). We further analyzed the relationship between the types of anesthesia and postoperative morbidity and mortality in patients receiving hip fracture surgery. Similarly, we found general anesthesia to be associated with higher risks of postoperative morbidity (adjusted HR, 1.17; 95% CI, 1.08–1.27) and mortality (adjusted HR, 1.13; 95% CI, 1.01–1.26) (Table 2).

#### 3.3.2. Multivariable Analysis after IPTW

After IPTW adjustment, general anesthesia remained associated with increased risks of postoperative adverse events (Table 2). In the IPTW multivariable Cox regression model, general anesthesia was associated with higher risks of postoperative adverse events (adjusted HR, 1.14; 95% CI 1.10–1.19), morbidity (adjusted HR, 1.19; 95% CI, 1.14–1.24), and mortality (adjusted HR, 1.09; 95% CI, 1.03–1.16).

Furthermore, we analyzed the relationship between general anesthesia and specific postoperative morbidities, including myocardial infarction, cardiac arrest, stroke, pneumonia, pulmonary embolism, ventilator support, acute renal failure or progressive renal insufficiency, surgical site infection, sepsis or septic shock, and DVT. The results revealed general anesthesia to be associated with an increased risk of cardiac arrest (adjusted HR, 1.23; 95% CI, 1.04–1.45), pneumonia (adjusted HR, 1.18; 95% CI, 1.09–1.27), ventilator support (adjusted HR, 1.42; 95% CI, 1.17–1.74), acute renal failure or progressive renal insufficiency (adjusted HR, 1.29; 95% CI, 1.10–1.51), surgical site infection (adjusted HR, 1.37; 95% CI, 1.20–1.56), sepsis or septic shock (adjusted HR, 1.32; 95% CI, 1.17–1.47), and DVT (adjusted HR, 1.38; 95% CI, 1.21–1.57) (Table 3).

#### 3.3.3. Propensity Score-Matched Analysis

Similar results regarding the relationship between general anesthesia and postoperative adverse events were found in the propensity score-matched cohort. In the propensity score-matched cohort, 9864 and 9864 patients received neuraxial and general anesthesia, respectively. After propensity score matching, all baseline characteristics of the two groups were well-balanced (Table 4). The relationship between general anesthesia and postoperative adverse events in the propensity score-matched cohort is presented in Table 5. The results of the multivariable Cox regression analysis revealed general anesthesia to be associated with a 25% increased risk of postoperative adverse events (adjusted HR, 1.25; 95% CI, 1.13–1.39). Further analysis demonstrated general anesthesia to be associated with a 30% higher risk of postoperative morbidity (adjusted HR, 1.30; 95% CI, 1.15–1.46). In addition, general anesthesia seemed to be related to a 19% increased risk of postoperative mortality (adjusted HR, 1.19; 95% CI, 1.00–1.42).

## 4. Discussion

The effects of different anesthesia techniques on postoperative outcomes in patients receiving hip fracture surgery remain unclear. Due to the small sample sizes in randomized clinical trials and the lack of clarity in definitions of postoperative outcomes in observational studies, previous meta-analyses have revealed no significant difference in postoperative outcomes between neuraxial anesthesia and general anesthesia in patients undergoing hip fracture surgery [9,15]. In addition, although DVT is a common postoperative complication of hip fracture surgery and antithrombotic prophylaxis is reportedly related to postoperative morbidity and mortality [16], no observational study has considered DVT prophylaxis in comparing the effects of different anesthesia techniques on postoperative complications. The present study collected data from the nationally verified ACS NSQIP database, which included clearly defined postoperative outcomes and data on DVT prophylaxis, to investigate and compare the associations of neuraxial and general anesthesia with postoperative adverse events after hip fracture surgery. To the best of our knowledge, this is the first large-scale, nationwide observational study investigating the association between different anesthesia techniques and postoperative outcomes in consideration of DVT prophylaxis. The results of the current study demonstrated that neuraxial anesthesia is associated with lower risks of postoperative complications, including postoperative morbidity and mortality. In addition, a similar relationship was found between neuraxial anesthesia and reduced postoperative complications, after adjustment for IPTW, and in the propensity score-matched cohort. Furthermore, our findings suggest that neuraxial anesthesia is associated with a reduced risk of cardiac arrest, pneumonia, ventilator support, renal failure, surgical site infection, sepsis or septic shock, and DVT.

The results of the present study reveal that general anesthesia is associated with an increased risk of 30-day mortality, which is in line with the findings of a previous observational study [17]. However, the results of previous randomized clinical trials showed that there was no significant difference in 30-day mortality between the two anesthesia groups [18,19]. The low postoperative mortality rate and the small number of included patients may be the reasons why these randomized clinical trials could not demonstrate differences in 30-day mortality between the two anesthesia techniques. Nevertheless, the results of a randomized trial involving 1600 older adults undergoing hip fracture surgery also showed that the incidence of postoperative mortality did not differ between patients who received neuraxial anesthesia and those who received general anesthesia [7]. In addition, our findings regarding mortality are inconsistent with those of previous observational studies that used propensity score matching, weighting, or stratification to control for confounders [20,21,22,23,24]. The inconsistency between the 30-day mortality results of the current study and the previous observational studies may be due to the different study populations and the different sources of research data; the present study collected data from the ACS NSQIP database based on patients’ medical charts, which would be different from those collected from insurance claims.

Compared with general anesthesia, the advantages of neuraxial anesthesia include the avoidance of intubation and mechanical ventilation, decreased systemic medications, prolonged postoperative analgesia, and decreased blood loss [15,25]. Conversely, general anesthesia may provide hemodynamic stability and avoid complications of neuraxial anesthesia, such as infection, hematoma, and nerve injury. Previous studies comparing the effects of general and neuraxial anesthesia on postoperative morbidity in patients receiving hip fracture surgery have reported conflicting results. The results of the two observational studies have demonstrated no difference in all-cause postoperative morbidity between patients who received general or neuraxial anesthesia for hip fracture surgery [22,23]. However, our findings reveal that general anesthesia is associated with higher all-cause postoperative morbidity. In addition to the composite outcome of postoperative morbidity, we further investigated the relationship between different anesthesia techniques and individual postoperative adverse events. The results of the present study reveal no significant differences between general and neuraxial anesthesia in the risks of postoperative 30-day myocardial infarction, stroke, or pulmonary embolism, which is consistent with the findings of most studies [12,22,23,26]. However, Ahn et al. [16] reported that general anesthesia was related to a higher incidence of pulmonary embolism than neuraxial anesthesia. Our findings additionally suggest that general anesthesia is associated with higher risks of postoperative 30-day surgical site infection and respiratory failure, which is consistent with the findings of previous studies [17,23]. Neuraxial anesthesia has been reported to reduce surgical site infections. This may be due to its effects on the sympathetic blockade and greater vasodilation, which lead to improved tissue oxygenation, increased polymorphonuclear cells at surgical sites, and maintained regional normothermia [27].

DVT is a common postoperative complication following hip fracture surgery and is associated with increased postoperative morbidity and mortality [16,28]. Unlike general anesthesia, neuraxial anesthesia can potentially produce a sympathetic block and vasodilatation, thereby reducing the risk of DVT [29]. With respect to DVT prophylaxis, our findings suggest that neuraxial anesthesia is associated with a lower risk of postoperative DVT, which is consistent with the findings of several studies [12,26,28,30]. However, the results of other observational studies and a meta-analysis of randomized clinical trials revealed no significant difference in the risk of DVT between general anesthesia and neuraxial anesthesia [15,22,23]. In addition, Morgan et al. [24] reported that patients who received spinal anesthesia were more likely to develop postoperative DVT. This inconsistency in the study results regarding postoperative DVT is likely due to the different study designs and definitions of outcomes.

The major strength of this study is that the data were collected from the ACS NSQIP, which is a nationally verified program for measuring and improving the quality of surgical care. The ACS NSQIP database contains data based on patients’ medical charts that were collected by trained and certified reviewers, which indicates that these data are highly trustworthy and different from those collected from insurance claims. In addition, all variables and outcomes in the ACS NSQIP database are clearly defined, which enhanced the accuracy of the results. Furthermore, the ACS NSQIP database has compiled data from more than 600 hospitals in the United States and around the world, thereby increasing its external validity. Finally, in addition to using IPTW to balance the measurable confounders between the two anesthesia groups, we used propensity score matching as a sensitivity test to evaluate the robustness of our findings.

Several limitations of the current study should be considered. First, although the data used in the current study were prospectively collected, patients were not randomized to the different anesthesia groups, which may have created a bias in our analysis. Even though we employed IPTW and propensity score matching to reduce bias, this bias cannot be eliminated. Second, extreme weights can increase the variance and confidence intervals of the effect estimate when using IPTW. However, there were no patients with a very high or very low probability of receiving general anesthesia. Third, the reasons why patients received general or neuraxial anesthesia and detailed clinical information regarding hospital-related factors, such as the size of the administering hospitals and the anesthesia and surgery techniques used for patients undergoing hip fracture surgery, were unavailable in the database, which may have also caused bias. Fourth, the ACS NSQIP collects postoperative outcomes for only up to 30 days; therefore, we were unable to evaluate the postoperative morbidity and mortality beyond that period. In addition, postoperative opioid consumption and adverse events immediately after surgery or in the postanesthesia care unit were not included in the ACS NSQIP database; thus, we were unable to analyze these outcomes.

## 5. Conclusions

In conclusion, the results of the present study suggest that, compared with general anesthesia, neuraxial anesthesia is associated with lower risks of postoperative adverse events in patients undergoing hip fracture surgery. Although the choice of the preferred anesthesia technique for hip fracture surgery remains controversial, the results of the present study support the administration of neuraxial anesthesia in hip fracture surgery.

## Figures and Tables

**Table 1:** Characteristics of patients receiving hip fracture surgery before and after adjustment for the inverse probability of treatment weighting (IPTW).

	Unweighted Study Population			After IPTW		
	Neuraxial Anesthesia	General Anesthesia		Neuraxial Anesthesia	General Anesthesia	
	(*n* = 9864)	(*n* = 36,010)				
Characteristics	Number (Percent of Patients)		SMD *	Percent of Patients		SMD *
Demographics						
Age, years						
<65	741 (7.5)	4422 (12.3)	0.160	12.3	11.3	0.033
65–74	1410 (14.3)	6407 (17.8)	0.095	16.8	17.0	0.007
75–84	3079 (31.2)	10,725 (29.8)	0.031	29.4	30.0	0.014
≥85	4634 (47.0)	14,456 (40.1)	0.138	41.6	41.7	0.002
Sex						
Female	6974 (70.7)	24,277 (67.4)	0.071	68.1	68.1	0.002
Male	2890 (29.3)	11,733 (32.6)	0.071	32.0	31.9	0.002
Race/ethnicity						
White	4807 (48.7)	28,447 (79.0)	0.664	72.7	72.5	0.003
Other	5057 (51.3)	7563 (21.0)	0.664	27.4	27.5	0.003
Body mass index						
Normal	4938 (50.1)	16,375 (45.5)	0.092	46.7	46.5	0.005
Underweight	926 (9.4)	2803 (7.8)	0.057	8.1	8.1	0.001
Overweight	2667 (27.0)	10,472 (29.1)	0.045	28.4	28.6	0.004
Obese	1333 (13.5)	6360 (17.7)	0.115	16.8	16.8	0.001
Functional health status						
Independent	7827 (79.4)	28,302 (78.6)	0.019	78.3	78.8	0.011
Partially dependent	1770 (17.9)	6764 (18.8)	0.022	19.1	18.6	0.012
Totally dependent	267 (2.7)	944 (2.6)	0.005	2.6	2.6	0.002
Comorbidities						
Smoking	1074 (10.9)	4585 (12.7)	0.057	12.7	12.3	0.011
Diabetes mellitus	1652 (16.8)	6986 (19.4)	0.069	19.3	18.9	0.012
Hypertension	6166 (62.5)	24,144 (67.1)	0.095	66.6	66.2	0.009
Congestive heart failure	297 (3.0)	1334 (3.7)	0.039	3.4	3.6	0.007
COPD	1120 (11.4)	3745 (10.4)	0.031	10.8	10.6	0.007
Dialysis	130 (1.3)	786 (2.2)	0.066	2.4	2.0	0.028
Dementia	2741 (27.8)	9501 (26.4)	0.032	26.9	26.7	0.004
Disseminated cancer	279 (2.8)	1306 (3.6)	0.045	3.6	3.5	0.008
Bleeding disorder	684 (6.9)	6884 (19.1)	0.368	16.1	16.5	0.013
Operative information						
Type of fracture						
Femoral neck fracture	4075 (41.3)	13,572 (37.7)	0.074	38.0	38.4	0.008
Intertrochanteric	5009 (50.8)	19,393 (53.9)	0.062	53.5	53.3	0.005
Subtrochanteric/other	780 (7.9)	3045 (8.5)	0.020	8.5	8.3	0.005
ASA classification						
I or II	1888 (19.1)	6001 (16.7)	0.065	18.0	17.3	0.017
III	5819 (59.0)	22,964 (63.8)	0.098	63.0	62.8	0.005
IV or V ^†^	2157 (21.9)	7045 (19.6)	0.057	19.1	19.9	0.022

* An SMD of less than 0.1 was considered a negligible difference between the two groups. ^†^ Includes 9119 (2130 received neuraxial anesthesia and 6989 received general anesthesia) ASA IV and 83 (27 received neuraxial anesthesia and 56 received general anesthesia) ASA V patients. Abbreviations: IPTW, inverse probability of treatment weighting; SMD, standardized mean difference; COPD, chronic obstructive pulmonary disease; ASA, American Society of Anesthesiologists.

**Table 2:** Association between general anesthesia and risk of postoperative 30-Day adverse events in patients receiving hip fracture surgery.

Postoperative 30-Day Outcomes	Total Number of Patients	Number of Events (%)	Unweighted Adjusted HR (95% CI) *	After IPTW Adjusted HR (95% CI) *
Any adverse events				
Neuraxial anesthesia	9864	1087 (11.0)	1.00 (reference)	1.00 (reference)
General anesthesia	36,010	4635 (12.9)	1.15 (1.07–1.23)	1.14 (1.10–1.19)
Morbidity				
Neuraxial anesthesia	9864	809 (8.2)	1.00 (reference)	1.00 (reference)
General anesthesia	36,010	3463 (9.6)	1.17 (1.08–1.27)	1.19 (1.14–1.24)
Mortality				
Neuraxial anesthesia	9864	437 (4.4)	1.00 (reference)	1.00 (reference)
General anesthesia	36,010	1951 (5.4)	1.13 (1.01–1.26)	1.09 (1.03–1.16)

* Adjusted HRs were computed after adjustment for age, sex, race or ethnicity, body mass index, functional health status, smoking, diabetes mellitus, hypertension, congestive heart failure, COPD, dialysis, dementia, cancer, bleeding disorder, type of fracture, ASA physical status classification, and DVT prophylaxis. Abbreviations: IPTW, inverse probability of treatment weighting; HR, hazard ratio; CI, confidence interval; COPD, chronic obstructive pulmonary disease; ASA, American Society of Anesthesiologists; DVT, deep venous thrombosis.

**Table 3:** Association between general anesthesia and risk of individual postoperative 30-Day morbidity in patients receiving hip fracture surgery.

Postoperative 30-Day Outcomes	Total Number of Patients	Number of Events (%)	Unweighted Adjusted HR (95% CI) *	After IPTW Adjusted HR (95% CI) *
Myocardial infarction				
Neuraxial anesthesia	9864	207 (2.1)	1.00 (reference)	1.00 (reference)
General anesthesia	36,010	722 (2.0)	0.98 (0.83–1.16)	1.03 (0.94–1.13)
Cardiac arrest				
Neuraxial anesthesia	9864	44 (0.5)	1.00 (reference)	1.00 (reference)
General anesthesia	36,010	264 (0.7)	1.39 (1.00–1.95)	1.23 (1.04–1.45)
Stroke				
Neuraxial anesthesia	9864	71 (0.7)	1.00 (reference)	1.00 (reference)
General anesthesia	36,010	288 (0.8)	1.06 (0.81–1.40)	0.95 (0.83–1.10)
Pneumonia				
Neuraxial anesthesia	9864	270 (2.7)	1.00 (reference)	1.00 (reference)
General anesthesia	36,010	1075 (3.0)	1.12 (0.97–1.29)	1.18 (1.09–1.27)
Pulmonary embolism				
Neuraxial anesthesia	9864	73 (0.7)	1.00 (reference)	1.00 (reference)
General anesthesia	36,010	285 (0.8)	1.11 (0.84–1.45)	1.00 (0.86–1.16)
Ventilator support ^†^				
Neuraxial anesthesia	9864	22 (0.2)	1.00 (reference)	1.00 (reference)
General anesthesia	36,010	202 (0.6)	1.84 (1.17–2.91)	1.42 (1.17–1.74)
Renal failure ^‡^				
Neuraxial anesthesia	9864	43 (0.4)	1.00 (reference)	1.00 (reference)
General anesthesia	36,010	284 (0.8)	1.49 (1.06–2.09)	1.29 (1.10–1.51)
Surgical site infection				
Neuraxial anesthesia	9864	89 (0.9)	1.00 (reference)	1.00 (reference)
General anesthesia	36,010	410 (1.1)	1.34 (1.05–1.71)	1.37 (1.20–1.56)
Sepsis or septic shock				
Neuraxial anesthesia	9864	104 (1.1)	1.00 (reference)	1.00 (reference)
General anesthesia	36,010	556 (1.5)	1.28 (1.03–1.60)	1.32 (1.17–1.47)
DVT				
Neuraxial anesthesia	9864	76 (0.8)	1.00 (reference)	1.00 (reference)
General anesthesia	36,010	411 (1.1)	1.38 (1.07–1.78)	1.38 (1.21–1.57)

* Adjusted HRs were computed after adjustment for age, sex, race or ethnicity, body mass index, functional health status, smoking, diabetes mellitus, hypertension, congestive heart failure, COPD, dialysis, dementia, cancer, bleeding disorder, type of fracture, ASA physical status classification, and DVT prophylaxis. ^†^ Ventilator support. ^‡^ Includes both acute renal failure and progressive renal insufficiency. Abbreviations: IPTW, inverse probability of treatment weighting; HR, hazard ratio; CI, confidence interval; DVT, deep venous thrombosis; COPD, chronic obstructive pulmonary disease; ASA, American Society of Anesthesiologists.

**Table 4:** Characteristics of patients receiving hip fracture surgery after propensity score matching.

	Neuraxial Anesthesia (*n* = 9864)	General Anesthesia (*n* = 9864)	
Characteristics	Number (Percent of Patients)		SMD *
Demographics			
Age, years			
<65	741 (7.5)	722 (7.3)	0.006
65–74	1410 (14.3)	1439 (14.6)	0.008
75–84	3079 (31.2)	3034 (30.8)	0.010
≥85	4634 (47.0)	4669 (47.3)	0.007
Sex			
Female	6974 (70.7)	6992 (70.9)	0.004
Male	2890 (29.3)	2872 (29.1)	0.004
Race/ethnicity			
White	4807 (48.7)	4830 (49.0)	0.005
Other	5057 (51.3)	5034 (51.0)	0.005
Body mass index			
Normal	4938 (50.1)	4948 (50.2)	0.002
Underweight	926 (9.4)	864 (8.8)	0.022
Overweight	2667 (27.0)	2706 (27.4)	0.009
Obese	1333 (13.5)	1346 (13.7)	0.004
Functional health status			
Independent	7827 (79.4)	7840 (79.5)	0.003
Partially dependent	1770 (17.9)	1772 (18.0)	0.001
Totally dependent	267 (2.7)	252 (2.6)	0.009
Comorbidities			
Smoking	1074 (10.9)	977 (9.9)	0.030
Diabetes mellitus	1652 (16.8)	1680 (17.0)	0.007
Hypertension	6166 (62.5)	6314 (64.0)	0.031
Congestive heart failure	297 (3.0)	275 (2.8)	0.012
COPD	1120 (11.4)	996 (10.1)	0.040
Dialysis	130 (1.3)	122 (1.2)	0.006
Dementia	2741 (27.8)	2848 (28.9)	0.024
Disseminated cancer	279 (2.8)	247 (2.5)	0.018
Bleeding disorder	684 (6.9)	665 (6.7)	0.006
Operative information			
Type of fracture			
Femoral neck fracture	4075 (41.3)	3993 (40.5)	0.017
Intertrochanteric	5009 (50.8)	5108 (51.8)	0.020
Subtrochanteric/other	780 (7.9)	763 (7.7)	0.006
ASA classification			
I or II	1888 (19.1)	1943 (19.7)	0.015
III	5819 (59.0)	5930 (60.1)	0.023
IV or V	2157 (21.9)	1991 (20.2)	0.042

* An SMD of less than 0.1 was considered a negligible difference between the two groups. Abbreviations: SMD, standardized mean difference; COPD, chronic obstructive pulmonary disease; ASA, American Society of Anesthesiologists.

**Table 5:** Association between general anesthesia and risk of postoperative 30-Day adverse events between propensity score-matched groups.

Postoperative 30-Day Outcomes	Total Number	Number of Events (%)	Unadjusted HR (95% CI)	Adjusted HR (95% CI) *
Any adverse events				
Neuraxial anesthesia	9864	1087 (11.0)	1.00	1.00
General anesthesia	9864	1254 (12.7)	1.16 (1.07–1.26)	1.25 (1.13–1.39)
Morbidity				
Neuraxial anesthesia	9864	809 (8.2)	1.00	1.00
General anesthesia	9864	939 (9.5)	1.17 (1.06–1.28)	1.30 (1.15–1.46)
Mortality				
Neuraxial anesthesia	9864	437 (4.4)	1.00	1.00
General anesthesia	9864	504 (5.1)	1.16 (1.02–1.32)	1.19 (1.00–1.42)

* Adjusted HRs were computed after adjustment for age, sex, race or ethnicity, body mass index, functional health status, smoking, diabetes mellitus, hypertension, congestive heart failure, COPD, dialysis, dementia, cancer, bleeding disorder, type of fracture, ASA physical status classification, and DVT prophylaxis. Abbreviations: HR, hazard ratio; CI, confidence interval; COPD, chronic obstructive pulmonary disease; ASA, American Society of Anesthesiologists; DVT, deep venous thrombosis.

## Data Availability

The data used in this study are from the American College of Surgeons National Surgical Quality Improvement Program (ACS NSQIP). Interested researchers can apply for the data by submitting a formal application to the ACS NSQIP.
